# Translation and cultural adaptation of evidence-informed leaflets on the work–health interface: a pragmatic approach to cultural adaptation

**DOI:** 10.1017/S1463423624000380

**Published:** 2024-10-11

**Authors:** Pål André Amundsen, Martin Engedahl, Kim Burton, Ira Malmberg-Heimonen, Margreth Grotle, Robert Froud

**Affiliations:** 1 School of Health Sciences, Kristiana University College, Oslo, Norway; 2 Professor of Occupational Healthcare, University of Huddersfield, Huddersfield, UK; 3 Department of Social Work, Child Welfare and Social Policy, Oslo Metropolitan University, Oslo, Norway; 4 Department of Rehabilitation Science and Health Technology, Oslo Metropolitan University, Oslo, Norway; 5 Warwick Clinical Trials Unit, Warwick Medical School, University of Warwick, Coventry, UK

**Keywords:** cultural adaptation, common health problems, health information material, translation/back-translation

## Abstract

**Aim:**

Our aim was to translate and culturally adapt three evidence-informed leaflets on the work–health interface from English into Norwegian. Integral to this aim was the exploration of the quality and acceptability of each of the adapted leaflets to Norwegian-speaking stakeholders; general practitioners, people who deal with health issues in the workplace, and the general population.

**Background:**

Common health problems, such as musculoskeletal pain, account for most workdays lost and disability benefits in Norway. To facilitate return to work, it may be important to have access to evidence-informed information on the work–health interface for stakeholders involved in sickness absence processes. However, there is limited information material available in Norwegian that is tailored for the different stakeholders. Cultural adaptation is an emerging strategy for implementing health information across different populations and regions. Guidelines on cultural adaptation are not well-suited for translating and adapting evidence-informed health information material.

**Methods:**

We conducted a pragmatic cultural adaptation process informed by existing guidelines. Our conceptual framework for adaptation is situated between adaptation and translation and comprises appraisal, forward- and back-translation, review in multiple steps, sense checking, and re-designing using a transcreation approach. Using an online survey, we aimed to evaluate the overall quality, value, acceptability, and clarity of each of the adapted leaflets to a total of 30 end-users.

**Findings:**

We translated and culturally adapted three leaflets from English to Norwegian. Adapted leaflets were found to be clearly presented, acceptable, and valued by 45 Norwegian end-users. No differences in key concepts between original and back-translated leaflets emerged through the review process by the original author and forward translators. We used a pragmatic approach in this study that might be useful to others culturally adapting evidence-informed health information material.

## Introduction

Common health problems, such as musculoskeletal pain, anxiety, and depression, and other mild psychological conditions, represent most of the long-term sick leave in Norway; defined as sick leave of eight consecutive weeks (Berg *et al*., 2021; NAV, 2022b). Of these, musculoskeletal pain is the most frequent cause of sick leave and work disability in Norway; accounting for 46% and 33%, respectively (Laerum *et al*., 2013; Clarsen *et al*., 2022). Disability benefits increased between 2011 and 2020, and the percentage of people outside the labour market increased between 2018 and 2022 (SSB, 2020; NAV, 2022a). It is known that being unemployed increases the risk of poor physical and mental health, while return to work (RTW) positively influences recovery in people with common health problems (de Vries *et al*., 2011; Xie *et al*., 2021).

Known obstacles to successful RTW include a lack of work-focused healthcare, challenges in implementing evidence, and poor communication between stakeholders, the worker, the employer, healthcare professionals, and the insurer (Frank *et al*., 1998; Loisel *et al*., 2005; Christian *et al*., 2006; Bartys *et al*., 2017; Lin *et al*., 2020). Targeted prevention using information material for health promotion and coordinating workplace-linked care systems are important to facilitate RTW (Frank *et al*., 1998; Kendall, 1999; Costello, 2016; McDaid *et al*., 2019). Providing evidence-informed information targeted to key stakeholders, emphasizing work-focused healthcare and the importance of communication, may help prevent work absence and facilitate RTW for people with common health problems. The Norwegian Labour and Welfare Administration (NAV) and other governmental institutions have several online resources about work and health available for use in Norway (Helsedirektoratet, 2018; NKARR, 2020; NAV, 2022c). However, these are either general in nature or focus on established schemes delivered by NAV. There is a need to translate and culturally adapt existing information material, which is tailored to key stakeholders (general practitioners [GPs], people who deal with health issues in the workplace, and the general population) in an RTW process.

### Cultural adaptation

Cultural adaptation refers to modifications made to material in order to make the material more suitable to a new target population with respect to culture, language, and context (Marshall *et al*., 2021). There is a growing body of literature on the cultural adaptation of measurement instruments (Perneger *et al*., 1999; Beaton *et al*., 2000; Acquadro *et al*., 2008; Sousa and Rojjanasrirat, 2011). Multiple guidelines exist for these, including the often-cited guideline from Beaton *et al*., (2000). Since the late 2000s, cultural adaptation of decision aids (DAs) has also received increased attention (Coudeyre *et al*., 2009; Ko *et al*., 2014; Berry *et al*., 2015; Jull *et al*., 2015; Tan *et al*., 2020). DAs are tools to help people to make appropriate clinical decisions for themselves (Whitney *et al*., 2004). Studies on cultural adaptation of these tools use various methods, likely due to the absence of appropriate guidelines (Chenel *et al*., 2018). The concept of ‘transcreation’ is now weighted within cultural adaptation; this asserts that it is not merely the translation and adaptation of the text that is important but also the infusion of culturally relevant context, photos, and themes (Díaz-Millón and Olvera-Lobo, 2021). The key is end-user utility and the underpinning maxim that ‘translation alone is not enough’ (House, 2006; Assaqaf, 2016; ECDC, 2016). The European Centre for Disease Prevention and Control (ECDC) has published a five-step guideline for cultural adaptation of health communication material, which includes transcreation features, such as appropriate design for the end-user population (ECDC, 2016). To our knowledge, at the time of writing, only one study has been conducted using the ECDC guideline, albeit with modifications (Baptista *et al*., 2020).

Our objectives were to translate and culturally adapt evidence-informed leaflets on the work–health interface for key stakeholders and explore whether these leaflets were thought to be of high quality, acceptable, and valued by (1) Norwegian general population, (2) Norwegian employers, line managers, and others in the workplace involved in work-health issues, and (3) Norwegian GPs.

## Methods

### Conceptual framework for adaptation

**Figure 1.**
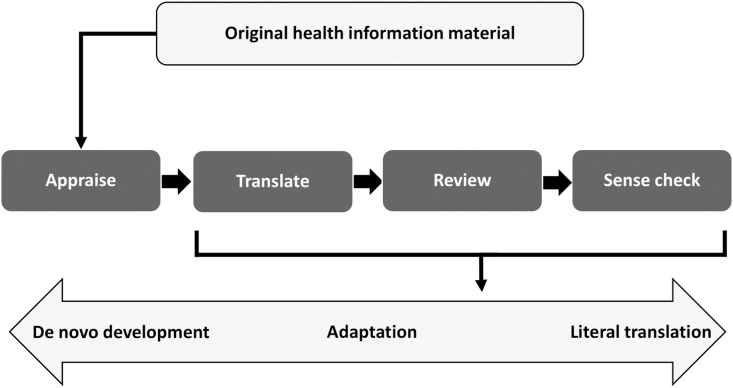
Cultural adaptation framework for existing health information material, adapted from Ko *et al*. (2014).

Our framework is based on guidelines for adapting health information (ECDC, 2016) and self-report measures (Beaton *et al*., 2000) and informed by the cultural and linguistic adaptation framework (Ko *et al*., 2014). Figure 1 shows our framework, which conceptualizes the adaptation process as locating an appropriate point along the spectrum between complete *de novo development* of health information material aimed at a specific population at one end and *literal translation* of existing material at the other. As the original leaflets are evidence-informed, our intentions were to preserve key messages and concepts, while at the same time respecting and making adaptations for cultural differences.

**Figure 2.**
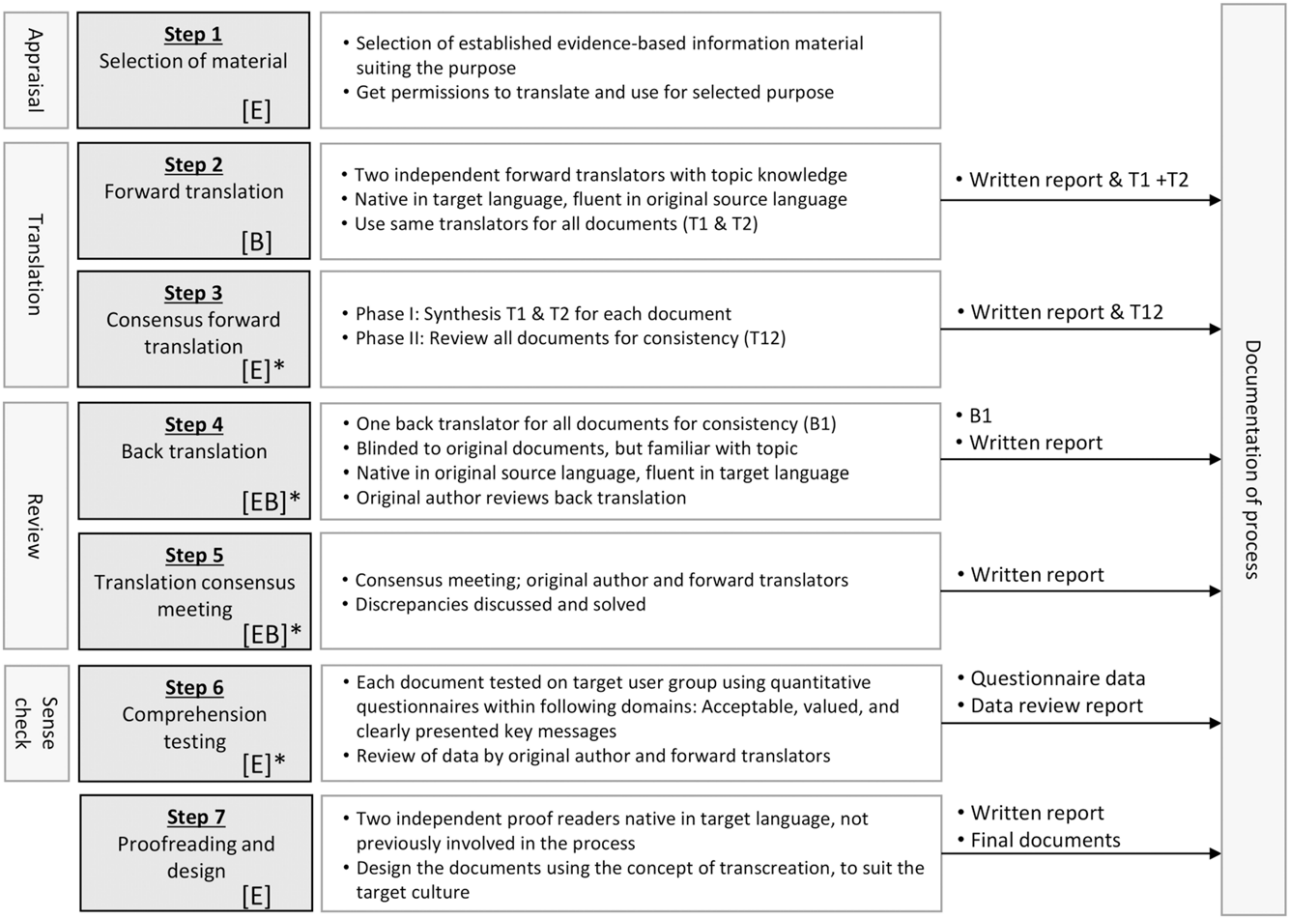
Stages involved in cultural adaptation of multiple leaflets, adapted from guidelines: [E] = ECDC, [B] = Beaton *et al*., [E]* = step adapted from [E], [B]* = step adapted from [B], and [EB]* = step adapted from both [E] and [B].

Our approach to comprehension testing involved testing the translated material’s overall quality, acceptability, and value to users, and whether key concepts were clearly presented (see ‘Sense checking’). Figure 2 shows the steps involved within our conceptual framework. Details of each involved step are elaborated in the text.

### Appraisal

#### Step 1) Selection of material

Within a Norwegian cohort randomized approach (ReISE) exploring RTW rates following individualised supported work placements with case manager support, the delivery of leaflets tailored to the participants, GPs, and line managers is an important part of the intervention (Froud, 2022; Amundsen *et al*., 2023). A set of leaflets is available in the UK, explaining the work–health interface (*i.e.*, the bidirectional relationship between work and health) and is tailored for GPs, people who deal with health issues in the workplace, and the general population (Anon, 2007a; Anon, 2007b; Anon, 2008). These three leaflets were used in a UK pilot of the ReISE intervention which was found to be acceptable and valued (Froud *et al*., 2020): (1) *Advising Patients About Work* (aimed at GPs); (2) *Work and Health* (aimed at people managing health issues in the workplace); and (3) *Health and Work* (aimed at the general population). These leaflets bridge a gap in Norwegian resources currently available and fit the requirements for the ReISE trial.

Originally, these leaflets were commissioned by the UK’s Department for Work and Pensions to support a Health, Work, & Well-being policy initiative. The starting point being an evidence review entitled ‘Is work good for our health and wellbeing?’ (Waddell and Burton, 2006) supplemented by a related review entitled ‘Concepts of rehabilitation for the management of common health problems’ (Waddell and Burton, 2004). Findings from these reviews were synthesised into a common set of messages on the work–health interface, along with practical information and advice, with the intention of facilitating a positive shift in the culture around work and health. The focus is supporting RTW and stay-at-work with common health problems. The synthesised material was used for the content of the leaflets and written in language to suit the three target groups. The leaflets were co-produced, whereby input on early drafts was obtained from stakeholder representatives, and the penultimate versions were then tested for comprehension and acceptability by additional stakeholders before the final versions were produced and published (K. Burton, personal communication, May 9, 2024).

We obtained permission to adapt the leaflets to use in the Norwegian ReISE trial. Original leaflets were commissioned by the UK Department for Work and Pensions and published by The Stationary Office (tso.co.uk). Kim Burton (KB), who was one of the original authors of the leaflets, is a collaborator on this work.

### Translation

#### Step 2) Independent forward-translation

The leaflets were reviewed and forward translated from English to Norwegian by two independent native Norwegian speakers, also fluent in English, and familiar with the topic (PA and ME). The aim was to ensure that culturally inappropriate advice and recommendations, as well as inapplicable myths, were omitted. Concepts, idiomatic words, or expressions that were difficult to translate into Norwegian were noted and alternative ways to convey their meanings were documented with the aim of achieving semantic equivalence in Step 3, while minimising material increases in word count.

#### Step 3A) Consensus forward-translation, Phase I

Consensus on translated versions of each leaflet was obtained using a two-phased approach; in Phase I, three consensus meetings were held, one for each leaflet. For each meeting, the two translated versions (T1 and T2) were reviewed by forward translators, and divergences were resolved by discussion. A third independent translator was consulted if divergences could not be initially agreed upon. The product of Phase I was a preliminary version of each translated leaflet.

#### Step 3B) Consensus forward-translation, Phase II

Phase II focused on consistency in concepts, idiomatic words, and expressions across the leaflets. The consensus meeting for Phase II included a review of all the leaflets from Phase I, making necessary adjustments to ensure consistency across leaflets. This step produced a final forward-translated version (T12) of each leaflet.

### Review

#### Step 4) Back-translation and review

Forward-translated leaflets (T12) were back-translated (B1) by an independent professional translator with experience in translating health material, a native speaker of English, and someone who was not familiar with the original source leaflets. One of the original authors of the leaflets (KB) then reviewed the back-translated leaflets and documented emerging differences in the key concepts, idiomatic words or expressions, and cases of unclear conceptual equivalence. A review report was sent to the forward translators prior to Step 5.

#### Step 5) Translation consensus meeting

Forward translators, and original authors, discussed the review report, compared B1 and original leaflets where necessary, and resolved any divergencies through discussion.

### Sense check survey

#### Step 6) Comprehension testing

We developed a feedback survey for each leaflet covering demographics, overall perceptions of quality, acceptability, and value, and whether key concepts were clearly presented. Survey items within the domains *acceptable* and *valued* were the same across the three surveys, with the exception of words related to the leaflets’ intended recipients and whether the leaflet gives the target user confidence/self-efficacy (*e.g.*, ‘knowing what I have to do to RTW’). To check whether the concepts were found to be clearly presented, each survey contained several statements about key concepts in the given leaflet.

### Primary survey outcome

Participants’ ratings of the provided leaflet’s overall quality, which was measured using an 11-point numeric scale with anchors from 0 (‘very bad’) to 10 (‘very good’) as a response option for the question ‘On a scale of 0–10, where 0 is very bad and 10 is very good, how would you rate this leaflet?’ We defined ratings over 5 to indicate ‘good’ perceptions of quality.

### Secondary survey outcomes

For secondary outcomes, we presented acceptability statements with two to three levels (*e.g.*, ‘Very easy to read’, ‘Quite easy to read’, and ‘Difficult to read’), and value statements (*e.g.*, ‘The leaflet makes me more confident in how to assist workers with common health problems to return to work’) on a 5-point scale ranging from 1 = ‘completely disagree’ to 5 = ‘completely agree’. A 5-point scale was also used for evaluation of clarity in key concepts, ranging from 1 = ‘very unclear’ to 5 = ‘very clear’ (*e.g*., ‘There are several myths and obstacles that can challenge the recovery. Identifying myths and obstacles are important to a worker’s recovery’).

### Sample size

The calculation of our sample size was based on being able to estimate mean ratings of leaflet quality to within a 95% confidence interval (CI) no greater than 2.5 units on the 11-point scale, which we reasoned sufficient to differentiate perceptions of good/and bad quality within each group. We assume a standard deviation of 2, which is typical in other studies (Kendrick and Strout, 2005; Kamper *et al*., 2009; Stjernberg-Salmela *et al*., 2022) and required a minimum of 10 participants from each leaflet’s user population to obtain a 95% CI of this width.

### Participants and recruitment

We used convenience sampling, recruiting through social media platforms and pain clinics, over a four-week period. Inclusion criteria were being employed as a GP (for ‘*Advising Patients About Work*’); having a role in dealing with health issues at the workplace such as line manager or within Human Resources (for ‘*Work and Health*’); or being of working age (for ‘*Health and Work*’). A link to an online survey tool (Nettskjema, UiO, Oslo) was provided with an information text to the relevant social media platform. Additionally, a flyer with a QR code to Nettskjema (see below) was created to recruit from pain clinics. Within Nettskjema, prior to answering the survey, a link to an external site containing the leaflet for review was provided.

#### Data collection and analysis

We used the online survey tool Nettskjema (UiO, Oslo) for data collection. Nettskjema has an embedded anonymisation function and personal metadata are not stored. Data collected in Nettskjema were exported to Stata 17 (StataCorp, Texas) for analysis using descriptive statistics and 95% CIs. Survey data were reviewed by the team, and potential issues were discussed.

#### Step 7) Proofreading and design

Proofreading was conducted by two independent native Norwegian speakers who had not read the leaflets before. Using the principle of transcreation (*vide supra*), the leaflets were re-designed with a new colour scheme in addition to new illustrations and pictures due to lack of availability of the original source files (Díaz-Millón and Olvera-Lobo, 2021).

## Results

As each leaflet was separately forward translated, this resulted in two versions for each leaflet to be compared. The Phase I consensus meeting revealed no difference in key concepts between the two forward-translated versions of the leaflets. However, several divergencies in the use of polysemic words, collocation, and structure of text appeared. Examples of the polysemic words that were considered include *work*, *support*, *accommodation*, and *recovery*, which depending on context, may have different meanings in Norwegian. One myth from the UK version, referring to the risk of getting fired if someone is excessively off sick, was omitted as the Working Environment Act in Norway protects the rights of employees being sick and as such this is not an extant belief (Lovdata, 2006). Within the Phase II consensus meeting, minor alterations were made to ensure consistency in key messages.

No major differences in key messages between the original source leaflets and back-translated leaflets emerged in the translation consensus meeting. Some terms were back-translated in a literal sense that gave rise to questions from the original author about the meaning, *e.g.*, the Norwegian word *hindring*, which was back-translated to *barrier* rather than the intended meaning which was *obstacles*. This is a key concept to translate appropriately in this context since the literature is careful to assert that obstacles, rather than barriers, exist and it is possible to navigate around those obstacles to RTW, as opposed to an artefactual barrier that stops progress for a person in their RTW. Other similar examples include *disease* versus *condition*, *risk* versus *consequence*, and *accommodate* versus *manage*. The back-translator tended to use a literal approach to these concepts which also required careful contextual consideration to retain fidelity to the messages in the original version. Thus, following the team discussion, no major changes to the Norwegian leaflets were made.

### Proofreading and design

The leaflets were proofread, and minor spelling mistakes were corrected. The format of the leaflets was kept as per the original, to facilitate typesetting, while the references were altered from in-text to providing a link to an online-reference site. Pictures and illustrations were considered culturally appropriate in the original version of all three leaflets but were changed to pictures with similar appearance due to lack of availability of the original source files. No data were specifically collected to evaluate the design of the leaflets.

### Sense check

During the four-week recruitment period, we received a total of 45 responses evaluating the proofread and re-designed leaflets. We received 10 responses from GPs who evaluated the *Advising Patients About Work* leaflet, 15 responses from people managing health issues in the workplace who evaluated the *Work & Health* leaflet, and 20 responses from the general population who evaluated the *Health & Work* leaflet.

**Table 1.** Respondent characteristics for the three leaflets^*^

	APAW (*n* = 10)	W&H (*n* = 15)	H&W (*n* = 20)
	*n* (%)	*n* (%)	*n* (%)
Gender			
Male	7 (70.0%)	8 (53.3%)	6 (30.0%)
Female	3 (30.0%)	7 (46.7%)	14 (70.0%)
Prefer not to specify	–	–	–
Age group			
<25	–	–	1 (5.0%)
25–34	1 (10.0%)	4 (26.7%)	3 (15.0%)
35–44	8 (80.0%)	7 (46.7%)	8 (40.0%)
45–54	–	1 (6.7%)	4 (20.0%)
54+ years	1 (10.0%)	3 (20.0%)	4 (20.0%)
Years of experience (for GPs, APAW leaflet)			
<5 years	6 (60.0%)	N/A	N/A
6–10 years	2 (20.0%)		
11–15 years	1 (10.0%)		
16–20 years	–		
>20 years	1 (10.0%)		
Work role (for workplace, W&H leaflet)			
HR (Human Resources)	N/A	3 (20.0%)	N/A
Line Manager		3 (20.0%)	
General Manager (CEO)		3 (20.0%)	
Section Leader		4 (26.7%)	
Occupational Health Service		1 (6.7%)	
Career Advisor		1 (6.7%)	
Insurer		–	
Solicitor		–	
Education (for general population, H&W leaflet)			
No qualification	N/A	N/A	–
Lower secondary school qualification			–
Upper secondary school qualification			5 (25.0%)
Vocational education (certificate/diploma)			4 (20.0%)
University level:			
Certificate or diploma			–
Bachelor’s degree			5 (25.0%)
Master’s degree			5 (25.0%)
PhD/other doctorate			1 (5.0%)

* Freely translated from Norwegian questionnaire.

APAW = Advising Patients About Work; assessed by GPs; W&H = Work & Health; assessed by people in the workplace; H&W = Health & Work; assessed by the general population.

### Demographics (Table 1)

The GPs were predominately male (70%) in their early careers, with eight of ten having less than 10 years of experience. Respondents from the workplace varied in terms of age and gender, but a majority had a manager or HR position (87%). Responders from the general population varied in terms of education and age and were predominately female (70%).

### Overall quality and key concepts

Each of the three leaflets was rated overall as ‘good’ on a scale from 0 (‘very bad’) to 10 (‘very good’). *Advising Patients About Work* targeted GPs and had a mean rating of 7.5 (95% CI 5.68 to 9.32) noting one outlier scoring the leaflet as 1. *Work & Health* targeted at people who deal with health issues at the workplace had a mean score of 8.0 (95% CI 7.40 to 8.59). *Health & Work* targeted at the general population had a mean score of 8.35 (95% CI 7.64 to 9.05).

Statements covering key concepts within the leaflets were generally reported as clear or very clear (Supplementary Material 1). The statement ‘Many obstacles to recovery exist; identifying and managing these obstacles is important for the outcome of rehabilitation’ in *Advising Patients About Work* revealed the lowest reported score among the leaflets, although still reportedly clear with a mean of 4.00 (SD 0.47) on a 0 (‘very unclear’) to 5 (‘very clear’) scale. For both the *Health & Work* leaflet and the *Work & Health* leaflet, all statements had a mean score of between 4.26 (SD 0.70) and 4.80 (SD 0.41), respectively, indicating that the key concepts were thought to be clear to very clear. The clarity of the statement ‘Work is generally good for health and wellbeing – including people with common health problems’ was rated highest in all leaflets, ranging from a mean value of 4.70 (SD 0.48) to 4.86 (SD 0.35).

### Acceptability and value

Combined, the leaflets were reported to be easy to read (86.6%), understand (100%), and to be of appropriate length (71.1%; Table 2). However, half of the GPs thought their leaflet was too long. Nearly all respondents thought the information was clearly presented (97.7%) and gave useful information (95.5%). Six out of ten GPs said they already knew most of the content, as did most people in the workplace (73.3%). On the other hand, most of the general population reported to have learned new and helpful information (70.0%). Almost all respondents suggested that the leaflet they evaluated should be given to the target population (93.3%).

**Table 2.** Acceptability and usefulness of Norwegian leaflets^*^

	Statements	APAW (*n* = 10)	W&H (*n* = 15)	H&W (*n* = 20)
		*n* (%)	*n* (%)	*n* (%)
1	Very easy to read.	9 (90.0%)	12 (80.0%)	18 (90.0%)
	Quite easy to read.	–	3 (20.0%)	2 (10.0%)
	Difficult to read.	1 (10.0%)	–	–
2	I understood all words and terms.	10 (100%)	15 (100%)	20 (100%)
	I understood most of the words and terms.	–	–	–
	Several words and terms were not understood.	–	–	–
3	The leaflet was too long.	5 (50.0%)	1 (6.7%)	5 (15.0%)
	The leaflet was too short.	–	2 (13.3%)	–
	The leaflet was of an appropriate length.	5 (50.0%)	12 (80.0%)	15 (75.0%)
4	I thought the information was clearly presented.	9 (90.0%)	15 (100%)	20 (100%)
	I thought the information was unclearly presented.	1 (10.0%)	–	–
5	I learned some new and helpful things.	4 (40.0%)	4 (26.7%)	14 (70.0%)
	I knew most of it anyway.	6 (60.0%)	11 (73.3%)	6 (30.0%)
6	I thought the leaflet gave useful information.	9 (90.0%)	15 (100%)	19 (95.0%)
	I did not think the leaflet gave any useful information.	1 (10.0%)	–	1 (5.0%)
7 **APAW**	General practitioners should read this leaflet.	8 (80.0%)	**N/A**	**N/A**
	It is not necessary for general practitioners to read this leaflet.	2 (20.0%)		
7 **W&H**	Those involved with workers health should read this leaflet.	**N/A**	15 (100%)	**N/A**
	It is not necessary for those involved with workers health to read this leaflet.		–	
7 **H&W**	The leaflet should be given to people with common health problems.	**N/A**	**N/A**	19 (95.0%)
	The leaflet should not be given to people with common health problems.			1 (5.0%)

* Freely translated from Norwegian questionnaire.

APAW = Advising Patients About Work; assessed by GPs, W&H = Work & Health; assessed by people in the workplace, and H&W = Health & Work; assessed by the general population.

The *Work & Health* and *Health & Work* leaflets were thought to provide important knowledge with mean ratings of 4.46 (SD 0.63) and 4.40 (SD 0.75), respectively, on a scale ranging from 0 (‘completely disagree’) to 5 (‘completely agree’) (Table 3). The *Health & Work* leaflet provided ‘confidence in work participation despite having a health problem’ with a mean rating of 4.15 (SD 0.87) and ‘knowing what to do to return to work’ with a mean rating of 4.2 (SD 0.83). The *Work & Health* leaflet gave ‘confidence on assisting workers return to work’, albeit with a slightly lower magnitude of 3.80 (SD 0.41). The *Advising Patients About Work* leaflet provided ‘knowledge and confidence in how to help this population’, with a mean rating of 3.70 (SD 1.05) and 3.90 (SD 0.87), respectively.

**Table 3.** Value of Norwegian leaflets on a 5-point scale (1 = completely disagree, 5 = completely agree)^*^

Leaflet	Statements	*n*	Mean (SD)	95% CI
APAW	The leaflet makes me more confident in how I can help these people.	10	3.90 (0.87)	2.94–4.42
	The leaflet gives important knowledge for general practitioners who meet these people.	10	3.70 (1.05)	3.27–4.52
W&H	The leaflet makes me more confident in how to assist workers with common health problems to return to work.	15	3.80 (0.41)	3.57–4.02
	The leaflet gives important knowledge into how the workplace can assist workers with common health problems.	15	4.46 (0.63)	4.11–4.82
H&W	The leaflet makes me more confident to participate in work despite having a health problem.	20	4.15 (0.87)	3.74–4.55
	The leaflet makes me more confident in knowing what I have to do to return to work.	20	4.20 (0.83)	3.81–4.59
	The leaflet gives important knowledge to people not currently working due to common health problems.	20	4.4 (0.75)	4.04–4.75

* Freely translated from Norwegian questionnaire.

APAW = Advising Patients About Work; assessed by GPs, W&H = Work & Health; assessed by people in the workplace, and H&W = Health & Work; assessed by the general population.

### Review of data

Team discussion following a review of the collected data included noting the potential future need to create an additional, less comprehensive version of the leaflet *Advising Patients About Work* for consumption by Norwegian GPs.

## Discussion

The culturally adapted and translated leaflets were considered to be of good quality. The *Work & Health* and *Health & Work* leaflets were considered acceptable and were valued by the target users with a clear presentation of key concepts. *Advising Patients About Work* was valued and clearly presented, albeit too lengthy according to half of the GP respondents.

Our work complements previous methodological studies on cultural adaptation, by providing a conceptual framework for a comprehensive, but low-cost process of culturally adapting evidence-informed health material. Our developed framework may be used as a guide to operationalise the adaptation process in similar cases (*e.g.*, in cases where existing evidence-informed health information material is required to be translated and culturally adapted). Conceptually, the described process involves the integration of a translation and back-translation process, consensus meetings, original author review, and operationalised through transcreation, with testing by target end-users. The translated and culturally adapted leaflets are aimed at stakeholders involved in an RTW process and may thus make a valuable contribution to Norwegian resources.

Other studies describing adaptations of health-related information material have used various methods (Coudeyre *et al*., 2003; Solomon *et al*., 2005; Coudeyre *et al*., 2009; Simmons *et al*., 2011; Ko *et al*., 2014; Jull *et al*., 2015; Berry *et al*., 2015; Chenel *et al*., 2018; Baptista *et al*., 2020; Tan *et al*., 2020). The common features are forward- and back-translation, with a qualitative approach to comprehension testing, using either in-depth interviews or focus groups. However, Coudeyre *et al*. (2003) used topic experts to ensure the quality of the translation in addition to questionnaire testing. Later, Coudeyre *et al.* (2009) considered forward- and back-translation sufficient for their adaptation of what was noted as a ‘simple’ decision-making tool (Coudeyre *et al*., 2009). Other studies have used interviews as part of exploring acceptability, usefulness and layout, or cultural relevance and how a decision process develops over time (Chenel *et al*., 2018).

There are several limitations to this study. Studies in the field of cultural adaptation most often use qualitative approaches to comprehension testing, as suggested by existing recommendations (ECDC, 2016; Beaton *et al*., 2000). Our approach to include target end-users is quantitative, without free-text responses, and the hazard is that any feedback users may have on nuances and interpretations in the adapted text or on design issues becomes impossible to report. However, as the original leaflets were evidence-informed and concisely written in order to be faithful to the evidence base, potential challenges to content might have occurred if we had chosen an interview approach, e.g. in cases where the evidence contradicted participants’ beliefs (Sinatra *et al*., 2014). Our approach, which consumes fewer resources than qualitative work to examine comprehension, may be sufficient when adapting between two similarly cultured countries when the underpinning evidence informing the original development of the leaflets has contributions from both countries. A contrasting example is the adaptation between North American people and Aboriginal people, where given major differences in culture and values, an approach such as ours would unlikely have been sufficient (Jull *et al*., 2015). The original leaflets and the evidence which they are informed by were mainly published between 2006 and 2008, which may suggest that an update of content (*i.e.*, key concepts) is overdue, notwithstanding location. However, evidence of the key concepts are still evident; for example, the benefits of work accommodations (Waddell *et al*., 2013; Cullen *et al*., 2018; Grant *et al*., 2019a), multidisciplinary efforts (Xie *et al*., 2021), and how coping strategies and RTW expectations influence work participation (de Wit *et al*., 2018; Grant *et al*., 2019b; Fisker *et al*., 2022). For the recruitment of people from the target populations, we used convenience sampling and small samples only in order to be able to separate to within 2.5 units of an 11-point scale, within the group. We caution against widespread generalisability due to the potential for under-representation given small non-random sampling (Etikan, 2016).

As we had overestimated the SDs in most cases, we were able to estimate within narrower CIs than planned. The developed framework and steps involved are comprehensive if a relatively crude sense-check (discriminating at least between perceptions of good and bad) of the adapted material is adequate. The approach then aligns with the aim of the comprehension testing according to ECDC; *i.e.* simply to know if the adapted and translated materials are clear and understandable to end-user groups for whom they are tailored (ECDC, 2016). As for the translation process, the ECDC does not specifically recommend the use of back-translators. However, with a few exceptions, most of the existing literature on adapting DAs and health information material have used back-translation (Simmons *et al*., 2011; Ko *et al*., 2014). We chose to use a back-translator and incorporated direct involvement from the original author to quality assurance and document the conceptual equivalence to the original work. To ensure consistency, we used the same translators for all leaflets. Had we used different translators for each leaflet, this might have introduced inconsistencies resulting from various use of words with similar, but not necessarily equivalent meanings, across the different leaflets, and additional resources would then be required to harmonise translated versions. As the translation process in itself is challenging, we caution that achieving perfect semantic equivalence may not be realistic (Eremenco *et al*., 2005). A moderately resource-intensive process, such as our described framework, may produce acceptable and useful results (Perneger *et al*., 1999). Using a multi-step approach with several consensus meetings helped ensure that differences in forward-translation approaches, and cultural issues, including mistakes and inaccuracies, were identified and addressed.

The general population and people dealing with health issues at work thought that their leaflet provided useful information on the relationship between health and work. Further research into the leaflets’ effects on work-related outcomes, such as RTW or stay-at-work rates, may be worthwhile (*i.e.*, as an inexpensive mini-intervention). All respondents from these two groups thought that the leaflet should be given to people with common health problems and to those involved with workers’ health, suggesting that these leaflets may fill a gap in currently available information material in Norway. Although a majority of GPs found their leaflet to provide useful information, they thought the leaflet was too lengthy, suggesting a revision being required for practical consumption by GPs, which might be considered as a future adaptation.

## Conclusion

Three leaflets were translated and culturally adapted from English and found to be of good quality, acceptable, and valued by Norwegian target users. Key concepts in the Norwegian leaflets were evaluated as conceptually equivalent to the original leaflets. In the absence of clear guidelines for the adaptation of evidence-informed health information material, our pragmatic approach may be able to be further refined and replicated by other workgroups to facilitate adding to the limited body of existing health information between countries with a common evidence base, and in a cost-effective manner.

## Supporting information

Amundsen et al. supplementary material Amundsen et al. supplementary material

## Supplementary material

To view supplementary material for this article, please visit https://doi.org/10.1017/S1463423624000380
