# Supplementary material for: Translation and cultural adaptation of evidence-informed leaflets on the work–health interface: a pragmatic approach to cultural adaptation
Source: Prim Health Care Res Dev. 2024 Oct 11;25:e42. doi: 10.1017/S1463423624000380 (PMC11569859; doi:10.1017/S1463423624000380)
Supplement: Amundsen et al. supplementary material [file S1463423624000380sup001.docx]

| **Supplementary material 1**  Clarity of key concepts and messages on a 5-point scale (1=very unclear, 5= very clear)* | | | |  |
| --- | --- | --- | --- | --- |
|  | **Statements from *Advising Patients About Work* (n=10)** | *n* | Mean (SD) | 95% CI |
| 1. | Work is generally good for health and wellbeing – including people with common health problems. | 10 | 4.70 (0.48) | 4.35 to 5.00 |
| 2. | To be in work is a health intervention and important for recovery. | 10 | 4.50 (0.52) | 4.12 to 4.87 |
| 3. | Many obstacles to recovery exist. To identify and manage these obstacles is important for the outcome of rehabilitation. | 10 | 4.00 (0.47) | 3.66 to 4.33 |
| 4. | It is important to discuss with the patient what they can still manage to do, rather than what they are not able to do. | 10 | 4.40 (0.51) | 4.03 to 4.77 |
| 5. | In collaboration with the patient and employer, the general practitioner is an important contributor in planning the return to work. | 10 | 4.40 (0.51) | 4.03 to 4.77 |
| 6. | Common health problems can often be accommodated at work with suitable adjustments and support. | 10 | 4.10 (0.31) | 3.87 to 4.32 |
| 7. | To guide a patient not to work due to common health problems can over time lead the health problem to be worse than what it originally was. | 10 | 4.10 (0.56) | 3.69 to 4.50 |
| 8. | It is possible to prevent much of the absence and disability due to common health problems. | 10 | 4.20 (0.42) | 3.89 to 4.50 |
|  | **Statements from *Work & Health* (n=15)** |  |  |  |
| 1. | Work is generally good for health and wellbeing – including people with common health problems | 15 | 4.86 (0.35) | 4.67 to 5.00 |
| 2. | The workplace has an important role in getting workers back to their usual job routine. | 15 | 4.60 (0.50) | 4.31 to 4.88 |
| 3. | Common health problems can often be accommodated on the workplace with appropriate adjustments and support, and by overcoming obstacles. | 15 | 4.46 (0.51) | 4.18 to 4.75 |
| 4. | There are several myths and obstacles that can challenge the recovery. To identify and manage myths and obstacles are important to a worker’s recovery. | 15 | 4.66 (0.48) | 4.39 to 4.93 |
| 5. | It is important that all the people involved work together and towards the same goal. | 15 | 4.60 (0.63) | 4.25 to 4.95 |
| 6. | Key phrases in helping an employee with common health problems are: information and advice, keeping in touch, facilitate early return to work, temporary adjustments in work, and open dialog. | 15 | 4.26 (0.70) | 3.87 to 4.65 |
|  | **Statements from *Health & Work* (n=20)** |  |  |  |
| 1. | Work is generally good for health and wellbeing – including people with common health problems. | 20 | 4.80 (0.41) | 4.60 to 4.99 |
| 2. | The longer you are off work, the lower the chances of getting back to work. | 20 | 4.65 (0.48) | 4.42 to 4.87 |
| 3. | Getting better depends on collaboration between the health care system, workplace, and you. | 20 | 4.65 (0.58) | 4.37 to 4.92 |
| 4. | To return to work requires your motivation. | 19 | 4.42 (0.50) | 4.17 to 4.66 |
| 5. | To find out what you are still able to do is important – then your employer might assist with temporary adjustments. | 20 | 4.35 (0.48) | 4.12 to 4.57 |
| 6. | It is important that all treatment you receive is aimed at helping you get back to work. | 20 | 4.30 (0.65) | 3.99 to 4.60 |
| 7. | There are several myths and obstacles that can challenge recovery. To identify and manage myths and obstacles are important to get better. | 20 | 4.30 (0.57) | 4.03 to 4.56 |
| 8. | It is important to make a specific and individualised return to work plan and stick to the plan. | 20 | 4.60 (0.50) | 4.36 to 4.83 |
| 9. | I have an understanding of how I can get better and return to work | 20 | 4.30 (0.65) | 3.99 to 4.60 |

* Freely translated from Norwegian questionnaire.
